# Ovarian Adenocarcinoma With Leptomeningeal Metastases

**DOI:** 10.7759/cureus.27577

**Published:** 2022-08-01

**Authors:** Angel Bayas, Alona Kondramashin, Sadia Waheeds, Marc A Swerdloff

**Affiliations:** 1 Neurology, Florida Atlantic University Charles E. Schmidt College of Medicine, Boca Raton, USA; 2 Marcus Neuroscience Institute, Boca Raton Regional Hospital, Boca Raton, USA

**Keywords:** units with normal range (uwnm), leptomeningeal carcinomatosis (lmc) cerebrospinal fluid (csf), central nervous system (cns), enhancement of multiple nerve roots (emnr), cranial nerves (cn), ct scan, pet scans, ovarian serum carcinoma (oc), magnetic resonance imaging (mri), lumbar puncture (lp)

## Abstract

Leptomeningeal carcinomatosis (LMC) is an uncommon disease that unfortunately has a rapid deterioration and a very poor prognosis with a devastating outcome. There has been an associated increase in the incidence of the leptomeningeal disease recently. There is a low percentage of LMC, around five percent of patients with metastatic disease. LMC has been presented in solid tumors such as breast cancer, lung cancer, melanoma, and GI malignancies. LMC is less likely reported in ovarian cancers. The clinical presentation of LMC is variable and will express according to where the cancer cells infiltrate. The malignant cells can travel with the cerebrospinal fluid (CSF) and deposit on the brain, cerebellum, spinal cord, cranial nerves, and spinal roots. We report this case as a clinical anatomical exercise for healthcare professionals.

## Introduction

Leptomeningeal carcinomatosis (LMC) is an uncommon disease. It has a poor prognosis and devastating outcome. The clinical presentation varies with the location of cancer cell seeding. The diagnosis is made by the detection of malignant cells in the cerebrospinal fluid (CSF) and contrast magnetic resonance imaging (MRI) of the neuraxis. It is recommended to combine both for the best diagnostic yield due to the low sensitivity of each procedure, lumbar puncture (55%) and MRI (70%) [1]. Without treatment, the median survival of a patient is four to six weeks [2]. The prompt recognition of new neurologic deficits is crucial for early diagnosis in patients with systemic malignancies. Treatments vary depending on the type of primary carcinoma; a multi-disciplinary team is optimal for treatment and management. Craniospinal radiotherapy may be used as a palliative treatment [3,4]. We report a case of LMC in a patient with stage IV metastatic ovarian cancer (OC) presenting with new focal neurologic deficits, malignant cells in the spinal fluid and MRI showing infiltration of multiple cranial nerves and the cauda equina.

## Case presentation

A 56-year-old female patient with metastatic ovarian serous carcinoma presented with dyspareunia and pelvic pain in May 2020. A computed tomography (CT) scan revealed ascites with omental infiltration and elevated CA 125. The patient underwent cytoreductive surgery and was categorized as pT3cNxM1a, stage IV. The patient received systemic therapy of carboplatin, paclitaxel, and bevacizumab followed by olaparib/bevacizumab maintenance and off-label chemo-holistic therapy. A positron emission tomography (PET)/CT scan in August 2021 revealed the progression of the disease with activity in the infraclavicular lymph nodes, chest wall, axilla, abdominal, and peritoneum (Table 1).

**Table 1:** Shows the cerebrospinal fluid chemistry and hematology. CSF: cerebrospinal fluid.

Patient spinal fluid findings		
Laboratory parameters	Patient values	Reference range
CSF glucose	Less than 5 mg/dl	Normal: 50-80 mg/dl
CSF protein	547 mg/dl	Normal: 15-40 mg/dl
CSF polymorphonuclear white count	71 cells/μl	Normal less than 1 cell/μl
CSF lymphocyte count	84 cells/μl	Normal less than 5 cells/μl

**Figure 1:**
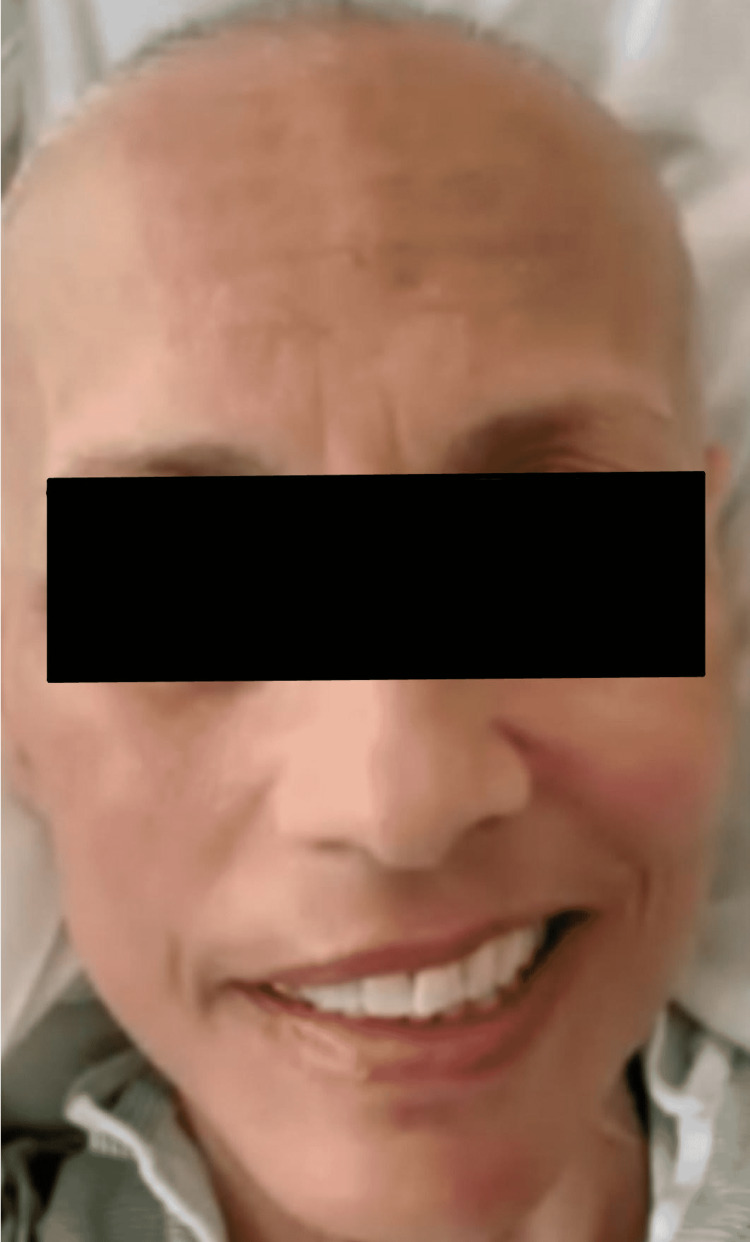
Right lower motor neuron facial nerve palsy is accentuated with a voluntary smile. Notice the flattened right nasolabial fold.

**Figure 2:**
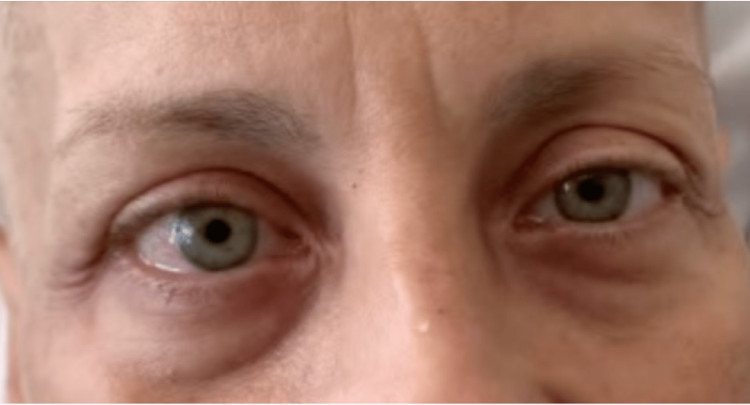
Esodeviation OD. The reflection from the window is obscuring the penlight reflection on the right iris. On the right gaze, there was a lag of abduction from a not fully recovered right sixth nerve paresis after a day of dexamethasone. OD: oculus dexter.

**Figure 3:**
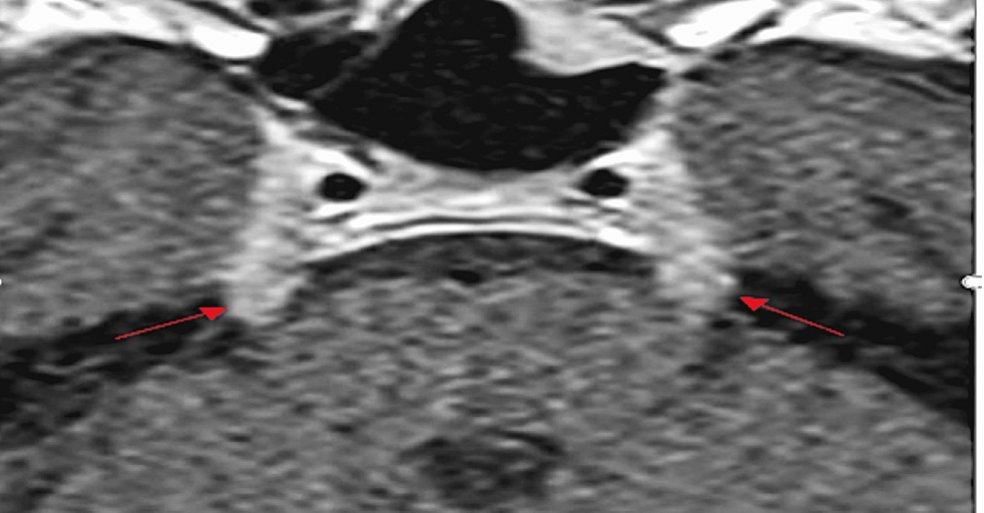
Bilateral thickened cranial nerve V (red arrows). MRI of the patient courtesy of Dr. Marc Swerdloff, August 2021.

**Figure 4:**
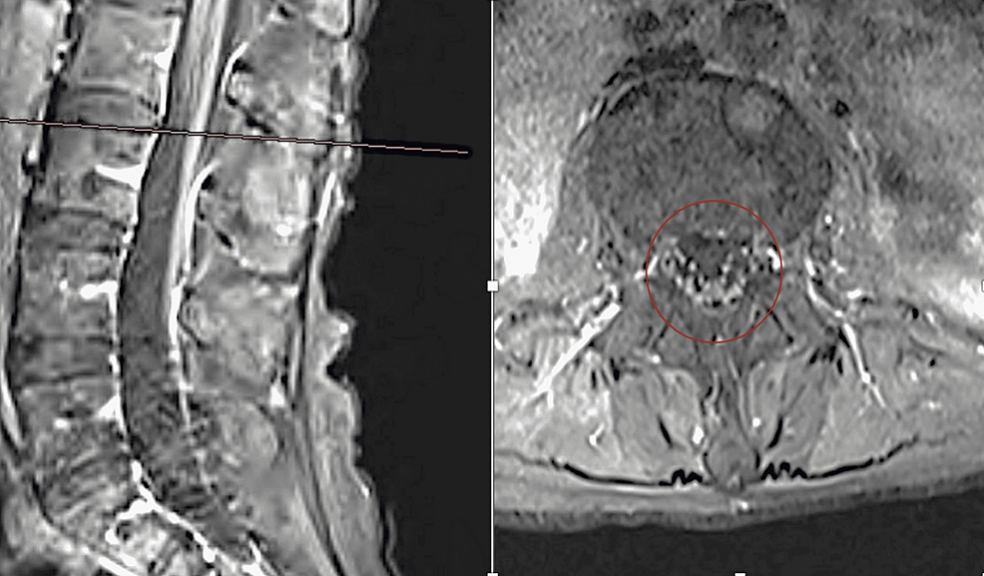
T1W contrast MRI lumbar spine with the enhancement of a thickened cauda equina and thickened enhanced conus medullaris. MRI of the patient courtesy of Dr. Marc Swerdloff, August 2021.

The patient presented to our hospital with progressive lower back pain, intermittent diplopia, difficulty swallowing, severe burning foot pain, and generalized weakness. Neurological examination revealed a right seventh cranial nerve palsy (Figure 1), a right six-nerve palsy with gaze paretic nystagmus (Figure 2), paraparesis, and decreased pinprick sense of the right foot and left foreleg. The patient was hypersensitive to light touch in a stocking distribution, which was from chemotherapy-induced neuropathy. Bilateral patellar and Achilles' tendon reflexes were absent. Starting oral dexamethasone increased her appetite and improved the right sixth nerve deficit. The patient had increased strength in her left leg but was still unable to raise it above gravity. MRI of the brain showed bilateral thickened cranial nerves V, VII, and VIII with enhancement and a right cranial nerve VI with enhancement but not thickened (Figure 3). The patient presents enhancement lesions on the lumbar spine with the enhancement of a thickened cauda equina and thickened enhanced conus medullaris (Figure 4).

A multidisciplinary team of gynecological oncology, neuro-oncology, radiation oncology, neurology and palliative care conferred with the patient to proceed with palliative radiation therapy to the brain and lumbar spine. She requested alternative holistic oncology treatment.

## Discussion

**Figure 5:**
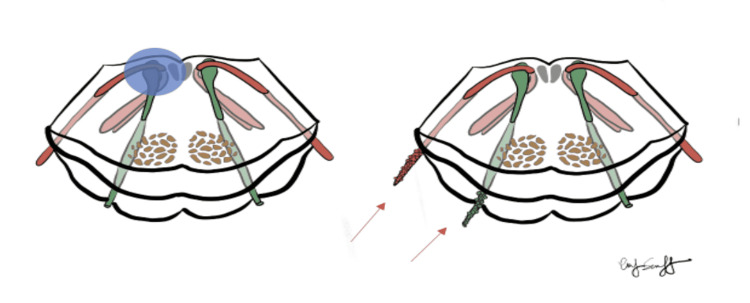
Close proximity in the brainstem of CN 6 and 7 (blue circle). Our patient with seeding of the individual cranial nerve fascicles that caused the equivalent clinical picture (red arrows). Illustration was done by Dr. Marc Swerdloff (co-author) in collaboration with Emily Swerdloff.

LMC is a rare condition. Ovarian LMC is also infrequent. The clinical presentation of LMC is variable since malignant cells can seed the entire neural axis wherever CSF flows [5]. Diagnosis may be challenging in this case. The differential diagnoses were tuberculous meningitis, bacterial, and viral meningitis. However, the patient did not present with headache, fever, or nuchal rigidity. After multiple attempts at a cure, our patient’s performance status declined rapidly. Initial MRI imaging failed to show evidence of intracranial or spinal cord disease. Our clinical examination pointed to multiple cranial nerves and spinal root involvement. Usually, an ipsilateral sixth and seventh cranial neuropathy suggests brainstem disease (Figure 5). 

**Figure 6:**
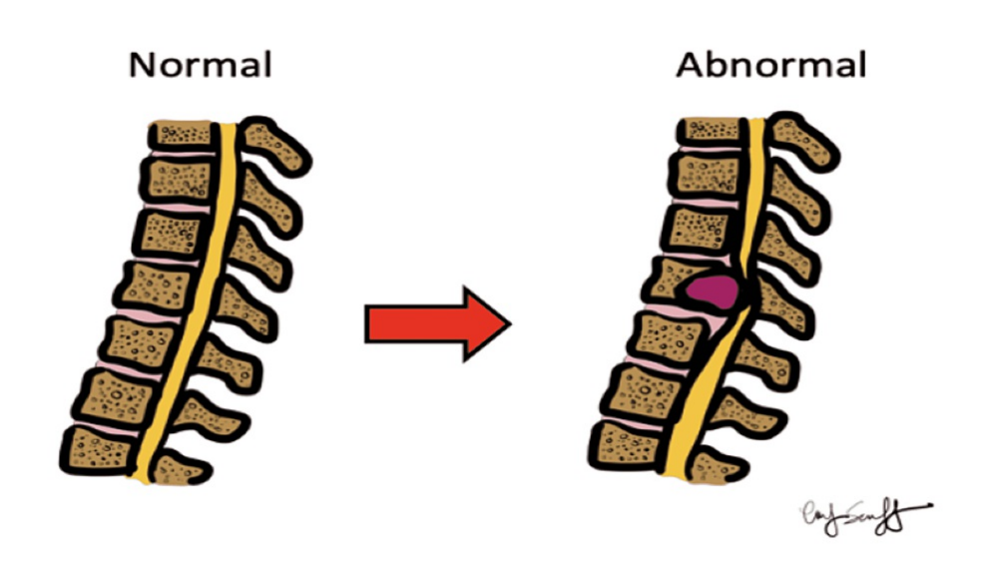
Typical cause of paraparesis in the patient with metastatic cancer. Illustration was done by Dr. Marc Swerdloff (co-author) in collaboration with Emily Swerdloff.

Paraparesis in the setting of advanced cancer and back pain usually indicates spinal cord involvement (Figure 6). 

**Figure 7:**
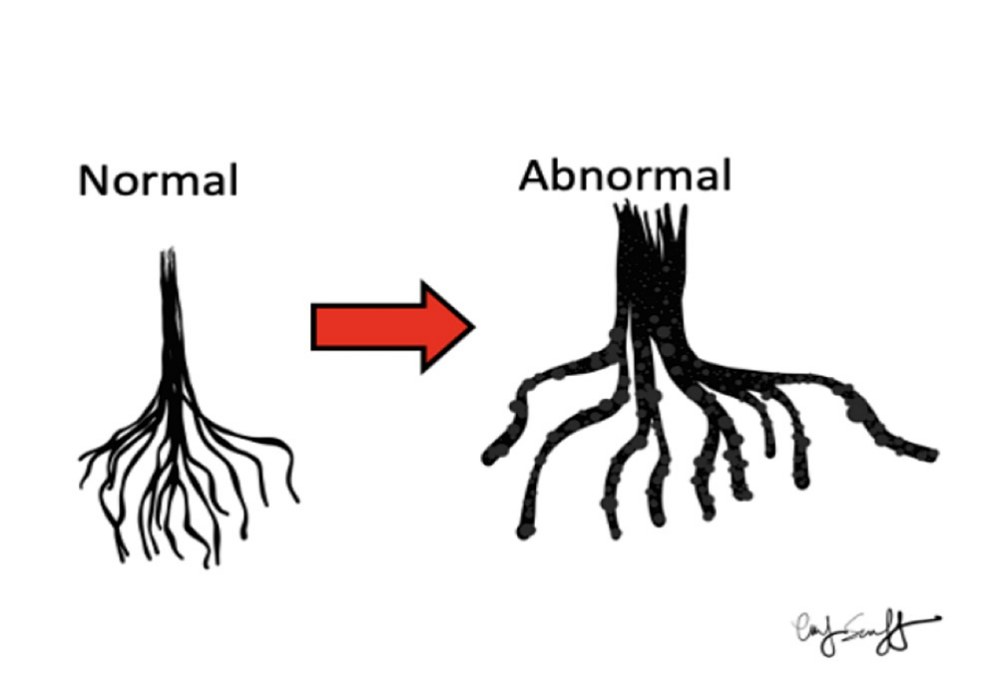
Leptomeningeal seeding of the cauda equina causing thickened roots. Illustration was done by Dr. Marc Swerdloff (co-author) in collaboration with Emily Swerdloff.

In our patient, seeding of the conus medullaris and lumbar roots caused paraparesis (Figure 7).

In the future, LMC will be more often encountered as therapeutic advances prolong the life of cancer patients. Similarly, improvements in diagnostic techniques and imaging will make the diagnosis easier [6-8]. Leptomeningeal metastases (LM) are usually a late manifestation of systemic disease and most often occur in patients after extensive therapy with surgery, radiation, and chemotherapy [9]. The most common presenting symptoms of LMC are related to increased intracranial pressure (headache, nausea, and vomiting) and gait disturbances. Nuchal rigidity is present in 15% of cases. Hemiparesis and aphasia are uncommon. Since LM may involve any part of the nervous system, it will present with a variety of symptoms [10]. A new onset of neurological symptoms in a cancer patient should prompt a thorough investigation of the entire neuraxis [11]. A high-clinical suspicion is crucial to achieving an early diagnosis.

LMC may occur by four mechanisms: meningeal seeding from pre-existing hemispheric central nervous system (CNS) metastases, direct extension from the subdural or epidural tumor, direct extension from cites outside but adjacent to the CNS, and hematogenous spread [12].

A definitive diagnosis of LM metastasis requires documenting malignant cells in the CSF. In one-third of patients, CSF cytology is non-diagnostic, requiring repeat CSF cytological examinations [12,13]. Diagnosis by non-contrast MRI or CT of the brain has been reported, but MRI with Gd contrast is more sensitive [14].

Treatment of LM metastasis remains palliative with the aim of preventing progression and improving quality of life. Typical treatment includes external beam radiation and systemic and intrathecal chemotherapy [15-17]. Our patient was offered whole brain radiation. There have been no established standard treatment guidelines due to the rarity of the condition. 

## Conclusions

Leptomeningeal metastasis from ovarian cancer is an extremely rare condition and has a very poor prognosis with a fatal outcome. Careful attention to new focal neurological deficits will allow one to anatomically locate the metastatic disease and follow up with imaging studies. The delay of the diagnosis can lead to the patient’s health deteriorating faster than it would with an earlier diagnosis. Multidisciplinary teams are necessary to provide therapy and thus improve the patient’s quality of life. There is a limited supply of treatments, which are also rarely effective. This reinforces the necessity for reporting these case studies to encourage the development of treatment guidelines for leptomeningeal metastasis disease.
